# A case of primary ovarian signet-ring cell carcinoma treated with S-1/CDDP therapy

**DOI:** 10.1186/s13048-020-00636-5

**Published:** 2020-03-21

**Authors:** Tadahiro Shoji, Ryosuke Takeshita, Tatsunori Saito, Takeshi Aida, Shunichi Sasou, Tsukasa Baba

**Affiliations:** 1 Department of Obstetrics and Gynecology, Iwate Medical University School of Medicine, 19-1 Uchimaru, Morioka, Iwate, 020-8505 Japan; 2 Department of Obstetrics and Gynecology, Hachinohe Red Cross Hospital, 2 Nakaaketo, Tamonoki, Hachinohe, Aomori, 039-1104 Japan; 3 Department of Pathology and Laboratory, Hachinohe Red Cross Hospital, 2 Nakaaketo, Tamonoki, Hachinohe, Aomori, 039-1104 Japan

**Keywords:** Ovarian cancer, Signet-ring cell carcinoma, S-1, CDDP

## Abstract

**Background:**

Primary ovarian signet-ring cell carcinoma is extremely rare, with only five recent case reports. Almost all reported cases of ovarian signet-ring cell carcinoma have been treated with TC therapy and none have reported regarding the use of S-1/CDDP therapy. We report a case of primary ovarian signet-ring cell carcinoma treated postoperatively with S-1/CDDP therapy.

**Case presentation:**

We describe a 55-year-old woman diagnosed with stage IB primary ovarian signet-ring cell carcinoma that was treated with S-1/CDDP therapy. Preoperative transvaginal ultrasonography and contrast-enhanced computed tomography (CT) revealed a solid tumor measuring 10 cm in diameter in the pelvis. The tumor marker levels were as follows: CA125, 41.6 U/mL; CA19–9, < 2.0 U/mL; and CEA, 2.2 ng/mL. Ovarian cancer was suspected, and total abdominal hysterectomy, bilateral salpingo-oophorectomy, and omentectomy were performed. The left ovary was enlarged to greater than fist-sized, and there was a small amount of clear yellow ascites. Histological examination of the left ovary led to the diagnosis of signet-ring cell carcinoma. Histological examination of the right ovary also showed the presence of a signet-ring cell carcinoma. After surgery, upper and lower gastrointestinal endoscopy and positron-emission tomography-CT were performed to search for a possible primary lesion, but none was found. The patient was diagnosed with primary ovarian signet-ring cell carcinoma with FIGO Stage IB (PT1b, NX, M0). As postoperative adjuvant chemotherapy, S-1/CDDP therapy (S-1120 mg/day/body × 14 days, CDDP 50 mg/m^2^ day 8, q 21 days) was administered for six cycles. There was no recurrence 27 months after the initial treatment.

**Conclusions:**

We considered S-1/CDDP therapy was effective for primary ovarian signet-ring cell carcinoma. This is the first case report of primary ovarian signet-ring cell carcinoma treated with S-1/CDDP therapy in the world.

## Background

Ovarian signet-ring cell carcinomas are mostly metastasis of cancers of digestive organs such as stomach and large bowel (so-called Krukenberg tumors) and account for approximately 2% of all ovarian cancers [1]. Primary ovarian signet-ring cell carcinoma is extremely rare, with only five recent case reports [2–6]. TC (paclitaxel/carboplatin) therapy is the standard postoperative adjuvant chemotherapy for epithelial ovarian cancer based on the results of GOG158 and AGO studies [7, 8]. Therefore, almost all reported cases of ovarian signet-ring cell carcinoma have been treated with TC therapy and none have reported regarding the use of S-1/CDDP therapy. Here, we report a case of primary ovarian signet-ring cell carcinoma treated postoperatively with S-1/CDDP therapy along with a literature review.

## Case presentation

**Fig. 1:**
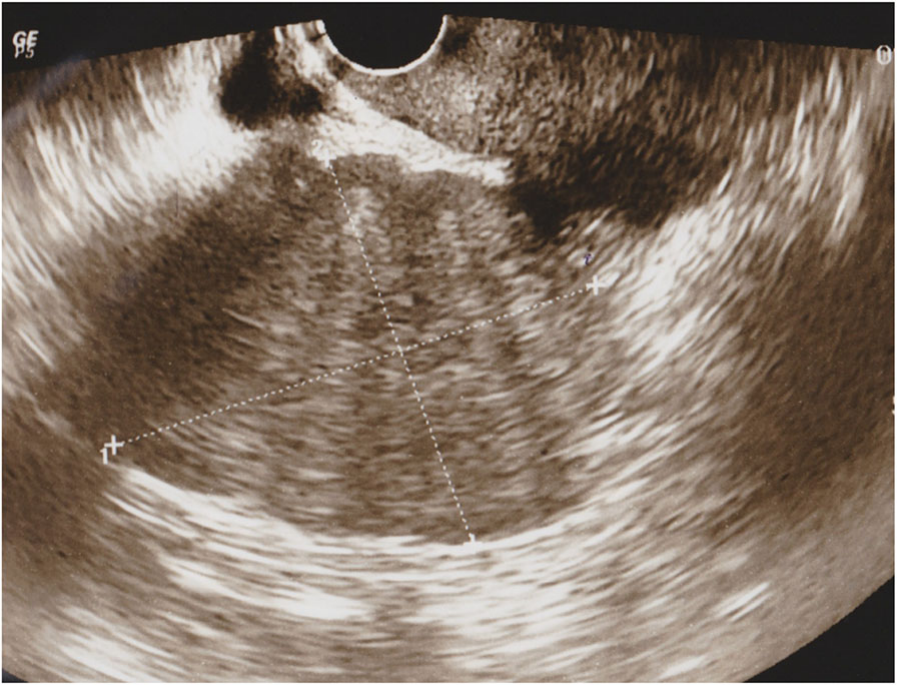
Transvaginal ultrasonography. A solid tumor margin measuring 10 × 8 cm in size is visible in the cranial part of the uterus

**Fig. 2:**
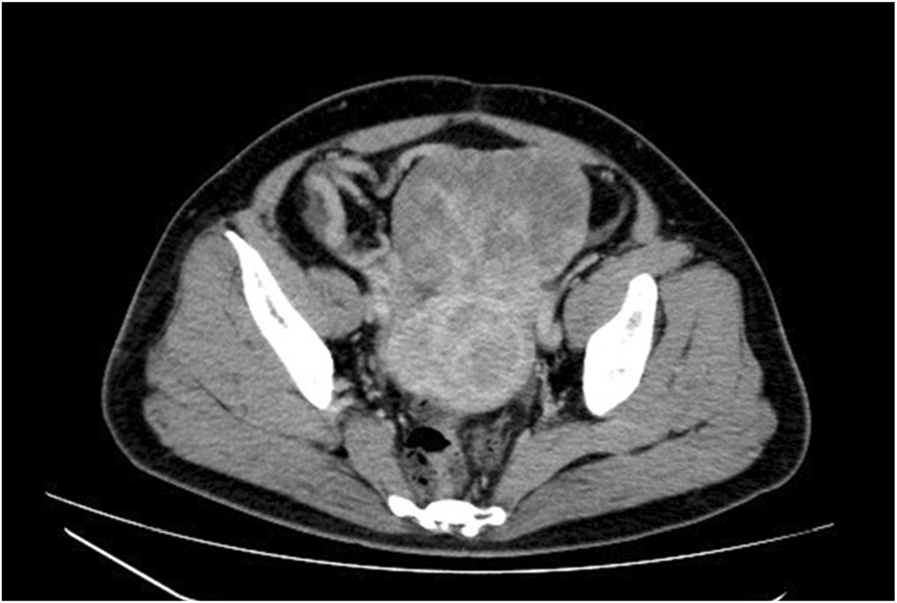
Contrast-enhanced computed tomography (CT) showing an irregularly enhanced solid mass measuring 10 × 8 cm in size in the cranial part of the uterine body, although the enhanced effect was lower. Lymph node enlargement suggestive of metastasis was not identified, and there were no metastases to other organs

**Fig. 3:**
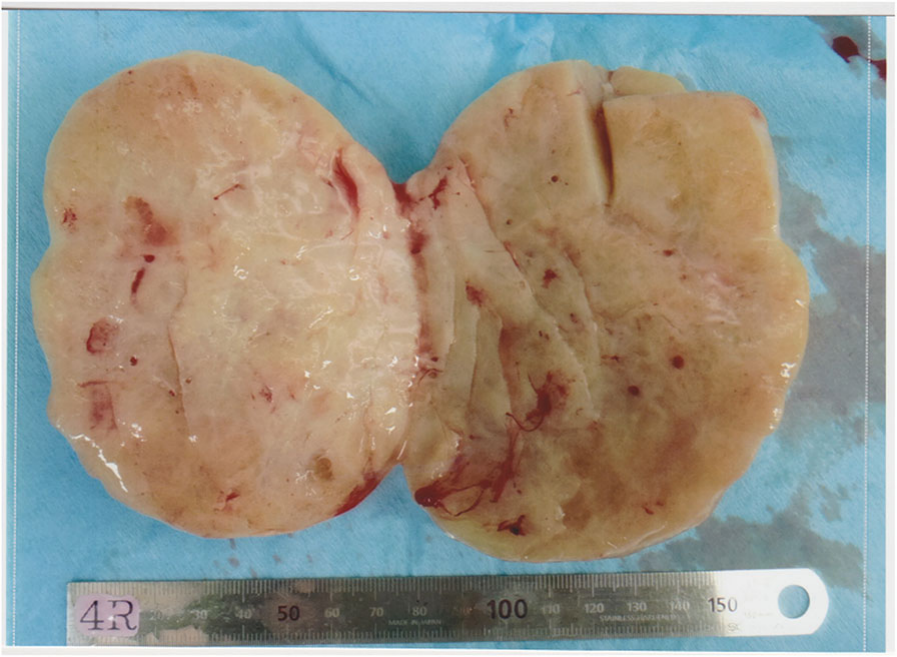
Macroscopic findings of the left ovarian tumor. The surface is smooth and pale yellow, and the cut surface is solid and pale yellow without mucus

**Fig. 4:**
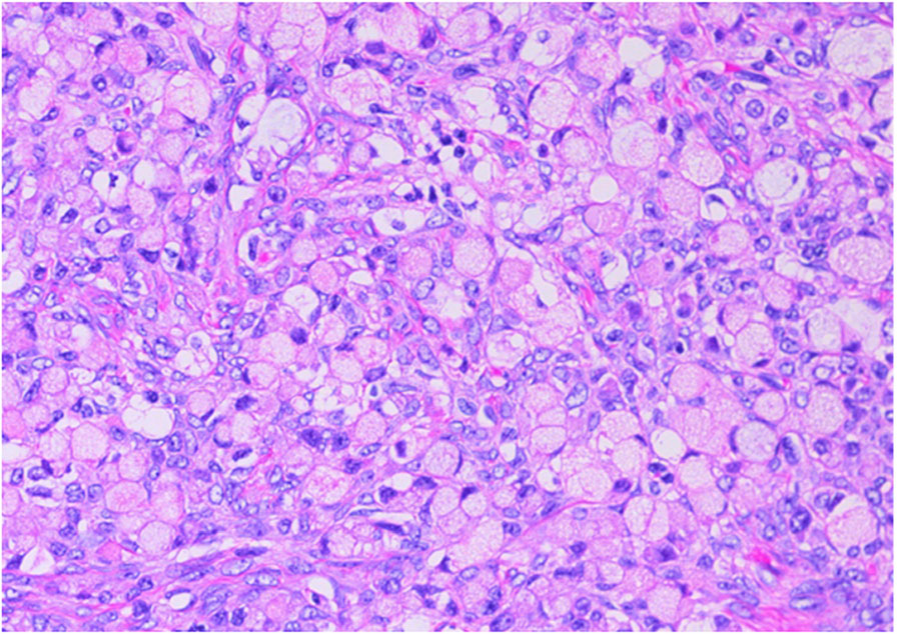
Hematoxylin and eosin (HE) staining showing solitary, sporadic, diffuse, and infiltrative proliferation of large, round-to- oval cells. These cells, in which the cytoplasm contained rich mucus and the nucleus was displaced toward one pole of the cell, were diagnosed as signet-ring cell carcinoma

**Fig. 5:**
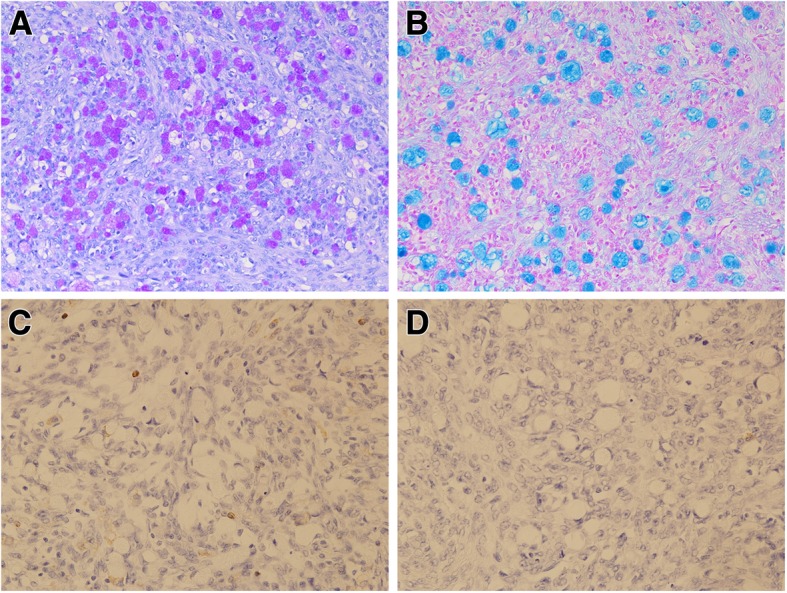
Positive staining for both periodic acid-Schiff (PAS) (**a**) and Alcian blue (**b**), however cytokeratin7(**c**) and 20(**d**) showed negativity

The patient was a 55-year-old woman, gravida 3, para 3. She had her first menstruation when she was aged 11 years and underwent menopause at 51 years of age. She was referred to our institution with a chief complaint of irregular vaginal bleeding; however, cytological examination of the uterine cervix and endometrium showed no abnormalities. At that time, the left ovary was solid and enlarged to 6 × 6 cm, but because there were no subjective symptoms and her CA125 level was 23 U/mL, the patient was followed up. Three months later, her left ovary had increased in size, and thus, surgery was planned. On pelvic examination, the uterus was the size of a goose egg, and a mobile mass greater than fist-sized was palpated in the cranial part of the uterus. Transvaginal ultrasonography showed a solid mass measuring 10 × 8 cm in size in this region (Fig. 1). Contrast-enhanced computed tomography (CT) also showed a solid mass measuring 10 × 8 cm in size in the cranial part of the uterine body (Fig. 2). Routine blood test and biochemical analysis results showed no abnormalities. Regarding tumor marker levels, CA125 levels were elevated at 41.6 U/mL, while CEA and CA19–9 levels were 2.2 ng/mL and < 2.0 U/mL, respectively, which were within the normal ranges. Magnetic resonance imaging was not performed because the patient had claustrophobia. She was diagnosed as having a solid ovarian tumor and thus underwent laparotomy. A small amount of clear yellow ascites was observed, and the left ovary was swollen to greater than fist-sized. The uterus was enlarged to the size of a goose egg, while the right ovary was thumb-tip in size. There was no obvious dissemination in the abdominal cavity. The rapid pathological diagnosis of the left ovary was signet-ring cell carcinoma. Total abdominal hysterectomy, bilateral salpingo-oophorectomy, and omentectomy were performed. Intraoperatively, the digestive tract was palpated by a surgeon, with no abnormal findings. Macroscopic examination of the extracted left ovarian tumor showed a smooth and pale yellow surface and a pale yellow and solid cut surface without mucus (Fig. 3). Cytology of the ascites was suspected to be positive. Histological examination (hematoxylin and eosin staining) showed a characteristic finding of signet-ring cell carcinoma with large and round-to-oval cells, in which the cytoplasm contained rich mucus and the nucleus was displaced toward one pole of the cell (Fig. 4). Both periodic acid-Schiff and Alcian blue staining were positive (Fig. 5a, b), cytokeratin (CK) 7 and CK 20 were negative (Fig. 5c, d). Based on these findings, the patient was diagnosed with signet-ring cell carcinoma. Histological examination of the right ovary also showed signet-ring cell carcinoma. As metastatic ovarian cancer was suspected, a whole-body examination was performed to search for a possible primary lesion. Upper and lower gastrointestinal endoscopy and positron-emission tomography-CT showed no findings suggestive of a primary lesion. Based on these findings, the patient was diagnosed with primary ovarian signet-ring cell carcinoma with FIGO Stage IB (2014) (PT1b, NX, M0). After surgery, S-1/CDDP therapy (S-1120 mg/day/body × 14 days, CDDP 50 mg/m^2^ day 8, q 21 days) was administered for six cycles after providing sufficient explanation and obtaining informed consent. The S-1/CDDP therapy for ovarian cancer was approved by the chemotherapy committee of Hachinohe Red Cross Hospital. The observed adverse events according to the Common Terminology Criteria for Adverse Events ver. 4.0 during the six cycles included grade 1 leukocytopenia, neutropenia, thrombocytopenia, liver dysfunction, nausea, vomiting, and leg edema. The six treatment cycles were completed without extending the length of the therapy or reducing the dose. Six months after the initial surgery, repeat upper and lower gastrointestinal endoscopy was performed, and no abnormalities were found. No recurrence was found 27 months after the initial surgery.

## Discussion

We experienced an extremely rare case of primary ovarian signet-ring cell carcinoma. The ovarian tumor was bilateral, and metastatic ovarian cancer was suspected. Therefore, after surgery, a whole-body examination, including gastrointestinal endoscopy, was performed to search for a possible primary lesion; however, there were no findings suggestive of a primary lesion. The rapid pathological diagnosis during surgery was signet-ring cell carcinoma, which was strongly suspected as metastasis of digestive cancer. Therefore, total abdominal hysterectomy, bilateral salpingo-oophorectomy, and omentectomy were performed without lymph node dissection.

Signet-ring cell carcinoma is a subtype of adenocarcinoma in which tumor alveolar foci of various sizes, mainly composed of signet-ring cells, are present in the hyperplastic interstitial connective tissue. Signet-ring cell carcinoma commonly originates from digestive organs, especially the stomach, but may also originate from the large bowel, pancreas, and appendix [3]. In 1896, Krunkenberg reported six cases of signet-ring cell carcinoma in which the primary lesions were assumed to be in the ovary [9]. In 1986, Vijay et al. reported a varying prognosis of primary ovarian signet-ring cell carcinoma, with some showing rapid progression ranging from several weeks to several months [10]. The survival rate of patients with signet-ring cell carcinoma is lower than that of patients with other histological types. The reasons for the poor prognosis are that signet-ring cell carcinoma displays a wide histological variety and is biologically malignant. Primary ovarian signet-ring cell carcinoma is infiltrative and is often accompanied by early lymph node metastasis, dissemination, and hematogenous metastasis, which are similar to the histological features of metastatic ovarian cancer. Moreover, the clinical course and prognosis of primary ovarian signet-ring cell carcinoma are similar to those of metastatic ovarian cancer.

S-1 is an oral anticancer drug that combines tegafur, a prodrug of fluorouracil, with 5-chloro-2,4-dihydropyrimidine (CDHP), and potassium oxonate at a molar ratio of 1:0.4:1. CDHP reversibly inhibits the activity of dihydropyrimidine dehydrogenase (DPD), the rate-limiting enzyme for the degradation of fluorouracil. Therefore, high concentrations of fluorouracil in serum and tumors are maintained for prolonged periods. Potassium oxonate blocks the phosphorylation of fluorouracil in the gastrointestinal tract, decreasing gastrointestinal toxic effects, the largest dose-limiting toxicity of fluorouracil [11]. S-1 is known to be active against gastric, colorectal, and pancreatic cancers [12–14]. On the other hand, cisplatin is generally believed to exert its anticancer effects by interacting with DNA, inducing programmed cell death [15].

Regarding the efficacy of initial chemotherapy for unresectable advanced/recurrent gastric cancer, the SPIRITS study compared S-1 monotherapy with S-1/CDDP therapy and reported a significantly extended median survival in the S-1/CDDP therapy group (13 months) compared with the S-1 monotherapy group (11 months) [16]. Based on this result, S-1/CDDP therapy has been recommended as the standard treatment for unresectable advanced/recurrent gastric cancer.

We administered S-1/CDDP therapy, the standard regimen for gastric cancer described above, as postoperative adjuvant chemotherapy to this patient. The standard regimen for unresectable advanced/recurrent gastric cancer consists of oral administration of S-1 (100 mg/day/body) for 21 days and intravenous administration of CDDP (60 mg/m^2^) on day 8, with a cycle of 5 weeks. However, the regimen used in the present case was the oral administration of S-1 (100 mg/day/body) for 14 days and intravenous administration of CDDP (50 mg/m^2^) on day 1, with a cycle of 3 weeks. The reasons for this change were as follows: the efficacy and safety of the regimen for unresectable advanced/recurrent cervical cancer have been demonstrated in a clinical trial mainly involving Japanese patients [17]; we had a considerable clinical experience with this regimen; and the dose intensity is increased with a 3-week cycle. In the SPIRITS study, the incidences of grade 3 or higher leukocytopenia, neutropenia, anemia, and thrombocytopenia were 11%, 40%, 26%, and 5%, respectively, and the incidences of nausea and anorexia were 11% and 30%, respectively [16]. In the study of S-1/CDDP therapy for unresectable advanced/recurrent cervical cancer by Aoki et al., the incidences of grade 3 or higher leukocytopenia, neutropenia, anemia, and thrombocytopenia were 32.4%, 52.7%, 34.6%, and 9.0%, respectively, and the incidences of nausea and anorexia were 3.2% and 12.8%, respectively [17]. In contrast, the adverse events in the present patient were minimal, with grade 1 leukocytopenia, neutropenia, thrombocytopenia, and liver dysfunction.

Table 1 shows a summary of previous studies of primary ovarian signet-ring cell carcinoma [2–6]. Some studies did not describe the disease stage or outcome, and chemotherapy was performed in only two studies. El-Safadi et al. reported a patient with stage IIIC primary ovarian signet-ring cell carcinoma who developed ascites immediately after completing TC therapy and had recurrence [3]. Ogawa et al. reported a patient with stage IIIC primary ovarian signet-ring cell carcinoma who died owing to the disease 2 months after receiving TC therapy [4]. We, therefore, expect that there were many patients who were diagnosed with ovarian signet-ring cell carcinoma with an unknown primary site and who were unsuccessfully treated with TC therapy. To our knowledge, this is the first reported case of primary ovarian signet-ring cell carcinoma that was treated with S-1/CDDP therapy in the world.

**Table 1:**
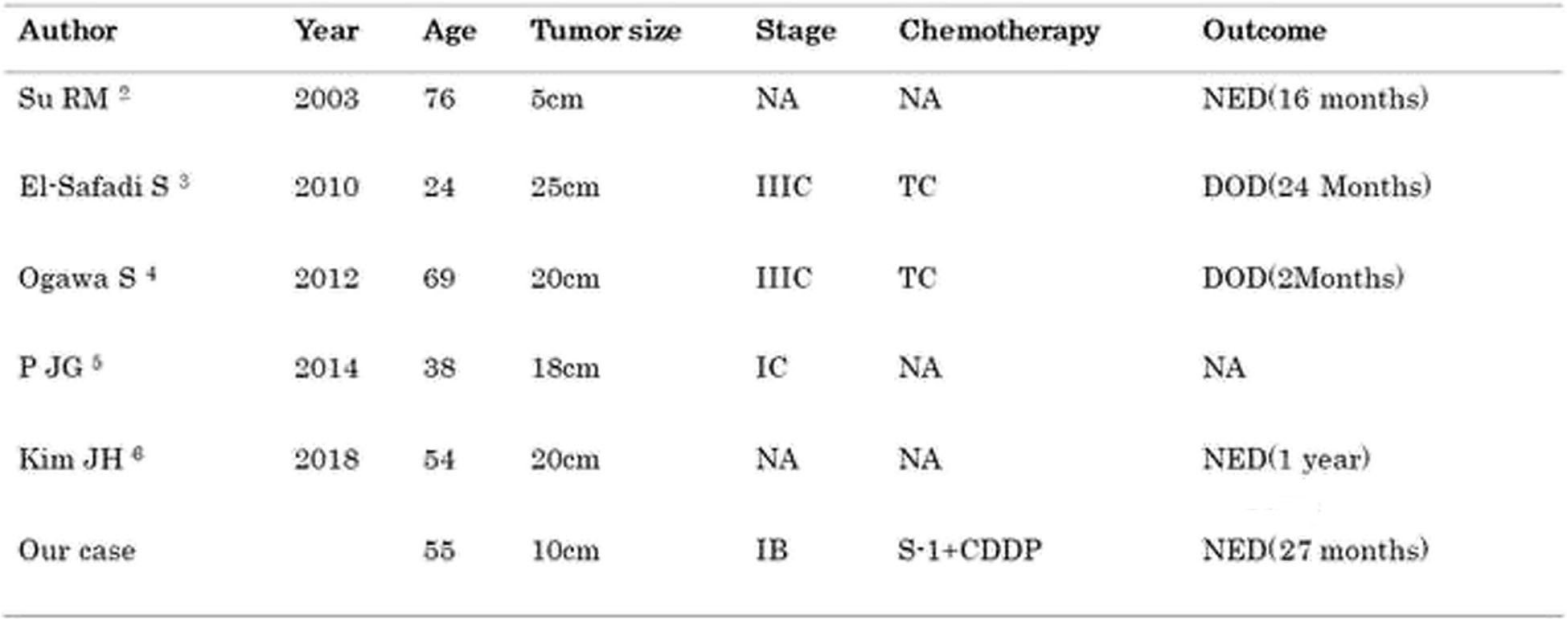
Previously reported cases with primary signet ring cell ovarian carcinoma

## Conclusions

We reported a case of primary ovarian signet-ring cell carcinoma treated with S-1/CDDP therapy. At the time of writing this report, the patient had no recurrence 6 months after completing S-1/CDDP therapy, suggesting that the therapy was effective. We expect that S-1/CDDP therapy can be a treatment option for patients with ovarian signet-ring cell carcinoma with an unknown primary site and that this strategy may be useful in clinical practice.
